# Different Expression Pattern of G Protein-Coupled Estrogen Receptor GPER1 in Esophageal Squamous Cell Carcinoma and Adenocarcinoma

**DOI:** 10.3390/ijms241814055

**Published:** 2023-09-13

**Authors:** Jingshi Liu, Yongdong Niu, Bin Zhang, Qisi Sun, Haiyi Li, Lu Bai, Zhongjing Su

**Affiliations:** 1 Department of Histology and Embryology, Shantou University Medical College, Shantou 515041, China; 2 Department of Pharmacology, Shantou University Medical College, Shantou 515041, China

**Keywords:** esophageal carcinoma, GPER1, estrogen, tissue microarray

## Abstract

Esophageal carcinoma is a male-dominant malignancy worldwide, and esophageal adenocarcinoma (EAC) shows more significant sex bias than esophageal squamous cell carcinoma (ESCC) in morbidity and mortality. The G protein-coupled estrogen receptor 1 (GPER1) is involved in several sex-related cancers; however, its expression level in esophageal carcinoma has been poorly investigated and its role is not precisely defined, depending on histological types. In the present study, the mRNA levels of GPER1 in esophageal carcinoma were collected from GEPIA and Oncomine databases for meta-analyses. The protein expression levels of GPER1 were detected by immunohistochemistry in the tissue microarray of EAC and ESCC. The GPER1 selective agonist G1, antagonist G15, and siRNA were applied in vitro to investigate their impacts on esophageal cell lines. Analysis of the RNA levels from the databases showed a decreased expression of GPER1 in overall esophageal carcinoma, and low expression levels of GPER1 were found to be associated with low survival of tumor patients. However, in the subgroup of EAC and its precancerous lesion, Barrett’s esophagus, overexpression of GPER1 RNA was increased when compared with the normal tissues. The average staining scores of GPER1 protein in the tissue microarray of EAC were significantly higher than normal esophageal samples, and the rate of positive staining increased with the grade of poor tumor differentiation. The scores of GPER1 protein in ESCC tissues were lower than those in the normal tissues. The results from cell line experiments in vitro showed that the GPER1 agonist G1 inhibited proliferation and promoted apoptosis of ESCC cells EC109 with positive expression of GPER1. G1 had no obvious effect on normal esophageal NE2 cells with weak expression of GPER1. In addition, GPER1 RNA knockdown and application of antagonist G15 reversed the effects of G1 on EC109. The results of this study indicate that the expression levels of GPER1 are higher in EAC than in ESCC, which might be correlated with the dimorphic estrogen signaling pathway in different types of esophageal carcinoma.

## 1. Introduction

According to the latest global cancer statistics information, esophageal carcinoma ranks seventh in terms of incidence and sixth in terms of being the cause of cancer-related mortality worldwide [1]. Esophageal carcinoma includes two main histologic subtypes, namely esophageal squamous cell carcinoma (ESCC), originating from the stratified squamous epithelia of the esophageal mucosa, and esophageal adenocarcinoma (EAC), arising from the columnar epithelia of the esophagus, which are quite different in pathology and epidemiology. The incidence of ESCC comprises over 90% of all esophageal cancer cases in low-income countries, and its occurrence is approximately 2–4 folds higher in men than in women. EAC is the most common type of esophageal cancer in several high-income countries [2,3]. Despite improvements in the diagnosis and treatment in recent decades, the overall 5-year survival rate of EAC remains less than 20% [4,5]. There are significant sex differences in the incidence of EAC worldwide, and the men-to-women ratio has been reported to be as high as 9:1 in the United States [6]. Multiple risk factors are correlated with the pathogenesis of esophageal cancer, and sex hormones and their signaling molecules are considered potential causes of this sex disparity.

Through a prospective population-based study, Petrick et al. found that high levels of circulating 17β-estradiol (E2) were associated with a reduced risk of EAC [7]. Moreover, postmenopausal women and younger women at menopause show a higher risk of developing EAC than those in the reproductive period [8]. Estrogen could mediate the physiological effects via gene regulation by nuclear estrogen receptors (ERs), including ERα and ERβ, which mainly function as transcription factors [9,10]. Notably, previous research on classic ERs and their signals in esophageal carcinoma was controversial. For ESCC, the expression levels of ERα and ERβ were inversely correlated, and the downregulation of ERα along with the upregulation of ERβ were responsible for the unfavorable prognoses of ESCC [11]. However, another meta-analysis showed that high ERα expression levels were associated with lower tumor differentiation and worse overall survival in ESCC, while ERβ overexpression was associated with favorable overall survival [12]. A variety of studies have found that ERβ is overexpressed in tumor tissues and associated with EAC lesion progression, whereas ERα is not expressed in EAC [13,14]. Nevertheless, Waleed Al-Khyatt et al. found no expression of either ERα or ERβ protein, but their mRNA expression levels were significantly higher in the tumor tissues [15]. These contradictory results suggest the possibility of other estrogen-signaling mechanisms associated with esophageal carcinoma.

GPER1, a member of the G protein-coupled receptor (GPCR) family, also known as GPR30, was first identified in 1996 in breast cancer tissues and was initially termed GPCR-Br [16]. Shortly afterward, studies discovered that this receptor was activated with E2 specifically and mediated a rapid non-genomic cell signal [17,18]. The expression patterns of GPER1 in various normal tissues have also been reported. It is also involved in estrogen-dependent diseases, including cancers of the reproductive system, cardiovascular system, and autoimmune diseases [19,20]. High expression levels of GPER1 were correlated with poor prognosis in patients with breast and ovarian cancers [21,22]. Low levels of GPER1 have been found to improve the survival of patients with cervical and endometrial cancers [23,24]. In addition to female-specific tumors, *GPER1* was also found to be a pathogenic gene in male prostate cancer [25]. In other male-dominant gastrointestinal tumors, the expression levels of GPER1 in gastric and colon cancers were lower than those in their corresponding normal tissues, and patients with low levels of GPER1 showed significantly poorer survival rates compared with those with greater levels of GPER1 [26,27].

As a novel membrane receptor for estradiol, GPER1 is also expressed in ESCC tissues [28]. However, the role of GPER1 in EAC remains unclear. To detect the role of GPER1 in different histological types of esophageal cancer, we examined the expression levels of GPER1 in esophageal cancer specimens by immunohistochemistry and followed these results by investigating the effect of GPER1 in esophageal cell lines in vitro via the treatment of specific agonist G1, antagonist G15, and knockdown of GPER1 expression by siRNA, thus providing a basis for analyzing the role of GPER1 in the sex bias and pathogenesis of esophageal malignancies.

## 2. Results

### 2.1. GPER1 mRNA Expression Was Decreased in Esophageal Cancer

**Figure 1:**
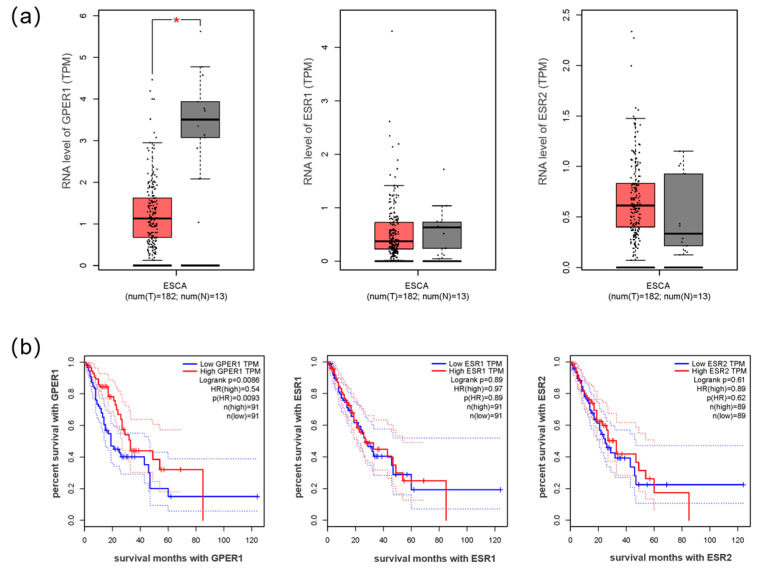
The RNA expression of estrogen receptors in esophageal cancer. (**a**) RNA levels GPER1, ESR1, and ESR2 in esophageal cancer compared with normal esophageal samples; (**b**) the survival curves of esophageal cancer with high and low expression of GPER1, ESR1, and ESR2 RNA. ESCA: esophageal carcinoma; T: tumor tissue of esophageal cancer (red box); N: normal tissue of esophagus (gray box); group cutoff: median. TPM: transcripts per million. HR: hazard rate. * *p* < 0.05.

A total of 182 esophageal cancer and 13 normal esophagus samples were retrieved from the GEPIA database. Analysis of the data showed that GPER1 mRNA levels were lower in the esophageal carcinoma tissues than the normal esophageal tissues (*p* < 0.05), and samples with low GPER1 (*n* = 91) RNA levels showed a lower survival rate than those with high levels (*n* = 91) of GPER1 RNA (*p* < 0.01). The expression levels of ESR1 and ESR2 RNA (encoding ERα and ERβ protein, respectively) in esophageal cancer were not significantly different from those in the normal esophageal samples (*p* > 0.05). There was no obvious relationship between the survival rate of the tumor patients and the RNA levels of ESR1 and ESR2 (*p* > 0.05) (Figure 1).

### 2.2. Barrett’s Esophagus Exhibited High Expression of GPER1 mRNA

**Figure 2:**
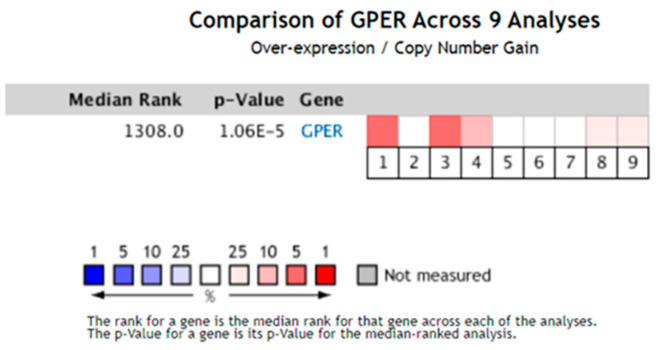
The expression levels of GPER1 mRNA were analyzed in nine cases of esophageal pathological and normal tissue from the Oncomine database. The square corresponding to each analysis indicates the gene rank, and the deeper red color indicates a higher gene rank and greater significance of over-expression. *p* < 0.05.

To further evaluate the expression levels of GPER1 in different histological subtypes of esophageal cancer, its mRNA levels were analyzed in nine cases of esophageal pathological and normal tissues. The results showed that GPER1 was over-expressed in esophageal pathological tissues in five analyses, of which three were affiliated with Barrett’s esophagus (*p* < 0.05), one with EAC, and one with ESCC (Figure 2).

### 2.3. GPER1 Protein Expression Levels Were High in EAC

**Figure 3:**
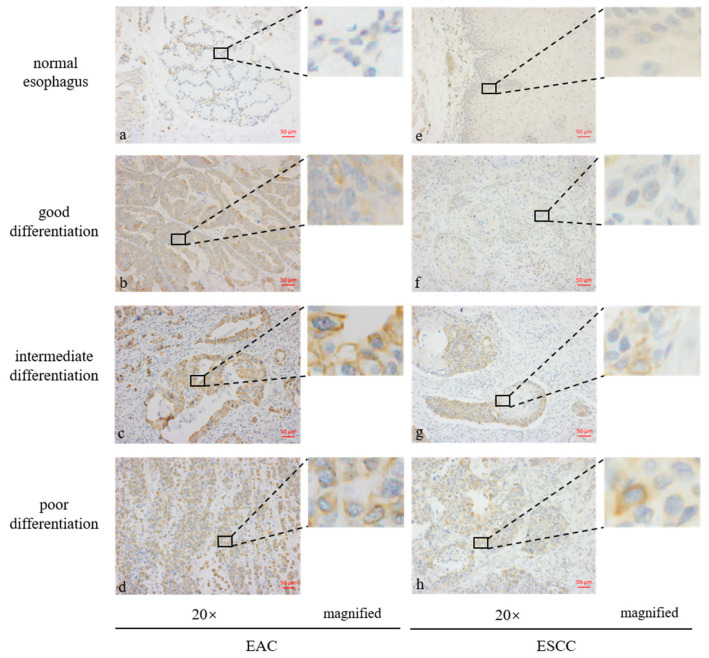
Expression levels of GPER1 protein in the tissue microarrays of esophageal tissues stained by immunohistochemistry. (**a**) Normal esophageal glands; (**b**) well-differentiated esophageal adenocarcinoma (EAC); (**c**) intermediately differentiated EAC; (**d**) poorly differentiated EAC; (**e**) normal stratified squamous epithelia of esophagus; (**f**) well-differentiated esophageal squamous cell carcinoma (ESCC); (**g**) intermediately differentiated ESCC; (**h**) poorly differentiated ESCC.

Staining the tissue microarray with the GPER1 antibody showed that the normal stratified squamous epithelia and glandular epithelia in most esophageal tissues were negative to weak staining. Positive staining was mainly located on the cell membrane of the tumor tissues. Representative immunohistochemistry staining of GPER1 protein is shown in Figure 3.

**Figure 4:**
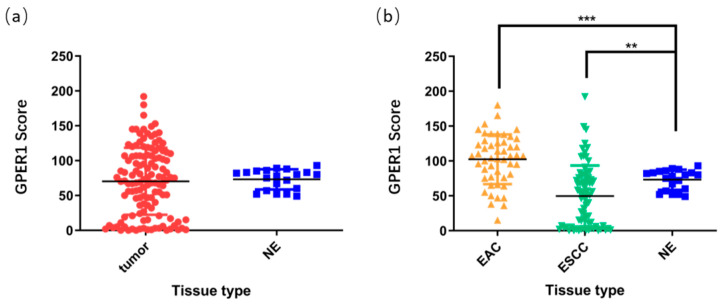
H-scores of GPER1 protein in the esophageal tissue microarray. (**a**) Overall esophageal carcinoma was compared to normal esophagus; (**b**) EAC and ESCC were compared to normal esophagus, respectively. NE, normal esophagus, *** *p* < 0.001; ** *p* < 0.01.

Evaluation of the H-scores for GPER1 in 148 tissue specimens using the Vectra Multispectral Imaging System demonstrated that there were no obvious differences between the overall cancer tissues and normal control tissues (*p* > 0.05, Figure 4a). After the esophageal cancer samples were subdivided into subtypes based on their histological features, EAC (*n* = 50) manifested higher expression levels of GPER1 (*p* < 0.001), while the expression levels in ESCC (*n* = 78) were relatively low compared with those in the normal esophageal samples (*n* = 20) (*p* < 0.01), as shown in Figure 4b.

### 2.4. Overexpression of GPER1 Was Associated with Poor Differentiation of EAC

According to the positive setting of H-scores ≥ 100, EAC showed 60% (30 out of 50) positive staining of GPER1 and ESCC showed 14.1% positive staining (11 of 78), and normal esophagus did not show any positive staining. The relationship between GPER1 protein expression levels and the clinicopathological characteristics of EAC is suggested in Table 1. Due to the limited number of cases with positive expression of GPER1 protein in ESCC, further analysis of the positive expression of GPER1 protein on the grade and stage of ESCC was not suitable. For EAC, we found that the positive expression of GPER1 protein increased with poor tumor differentiation (*p* < 0.05). Moreover, positive expression of GPER1 was more easily observed in stage I and IV EAC (*p* < 0.05). However, there is no significant difference in the expression of GPER1 among different ages, sexes, tumor depths, or lymph nodes (*p* > 0.05).

### 2.5. GPER1 Inhibited the Proliferation of Esophageal Carcinoma Cells

**Figure 5:**
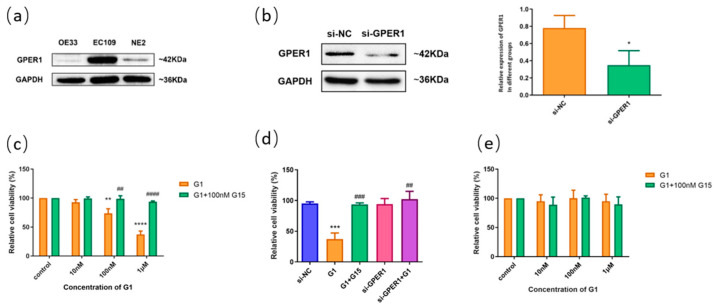
The effect of GPER1 on the cell viability of esophageal cells. (**a**) The protein levels of GPER1 were detected by Western blotting in OE33, EC109, and NE2 cell lines; (**b**) knockdown of GPER1 expression with siRNA in EC109 cell line, and the relative gray value of GPER1 to GAPDH; (**c**) the relative cell viability detected by MTT assay after EC109 cells were treated with different concentrations of G1 and G1 + G15; (**d**) the relative cell viability after EC109 cells were treated with si-NC, si-GPER1, and si-GPER1 + 1 μM G1, respectively; (**e**) the relative cell viability after NE2 cells were treated with different concentrations of G1 and G1 + G15. The results are shown as the mean ± SEM. * *p* < 0.05, ** *p* < 0.01, *** *p* < 0.001, **** *p* < 0.0001 vs. control/si-NC group; ^##^ *p* < 0.01, ^###^ *p* < 0.001, ^####^ *p* < 0.0001 vs. G1 group.

We detected the expressions of GPER1 in ESCC cell lines of EC109, EAC cell line OE33, and normal esophageal cell line NE2 using Western blot. The expression of GPER1 was weak in OE33 and NE2 cell lines (Figure 5a). Based on the expression level of GPER1 in the cell lines, we applied EC109 to explore the function of GPER1 through RNAi (Figure 5b) and receptor modulation. When the GPER1-positive expression cells EC109 were treated with GPER1 agonist G1 at 10 nM, 100 nM, and 1 μM for 48h, respectively, the cell proliferation detected using MTT assay showed that the proliferation of EC109 cells was significantly inhibited with the increase in G1 concentration. We expanded the research by using 100 nM GPER1 specific antagonist G15 to block the action of G1 and found that the proliferation of EC109 cells did not change significantly with the G1 concentration (Figure 5c). To confirm that GPER1 takes part in the regulation of cell proliferation caused by G1, we knocked down the GPER1 protein with GPER1 siRNA and then treated the cells with 1 μM G1. There were no statistical discrepancies between the GPER1 siRNA-transfected group and siRNA negative control group in the EC109 cell line (Figure 5d). For the GPER1-weak expression cells NE2, G1 treatment did not manifest an obvious change in the proliferation (Figure 5e).

### 2.6. The GPER1 Promoted Apoptosis of EC109 Cells

**Figure 6:**
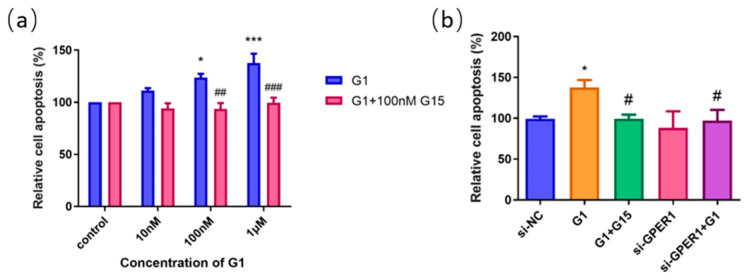
The effect of GPER1 on the cell apoptosis of EC109 cells detected by flow cytometry. (**a**) The relative cell apoptosis after EC109 cells were treated with different concentrations of G1 and G1 + G15; (**b**) the cell apoptosis after EC109 cells were treated with si-NC, si-GPER1, and si-GPER1 siRNA + 1 μM G1 group. The results are shown as the mean ± SEM. * *p* < 0.05, *** *p* < 0.001 vs. control/si-NC group; ^#^ *p* < 0.05, ^##^ *p* < 0.01, ^###^ *p* < 0.001 vs. G1 group.

To further investigate the effect of G1 on the apoptosis of EC109 cells, we treated cells with GPER1 agonist G1 at 10 nM, 100 nM, and 1 μM for 48 h, respectively, and the rate of cell apoptosis was detected by flow cytometry. The results showed that the apoptosis of the EC109 cell line was significantly promoted with the increase in G1 concentration, and the apoptosis induced by G1 was inhibited by the addition of 100 nM G15 (Figure 6a). In addition, cell apoptosis did not show obvious change for the GPER1 RNAi knocked down only (Figure 6b).

### 2.7. E2 Inhibit the Proliferation of EC109 Cells through GPER1

**Figure 7:**
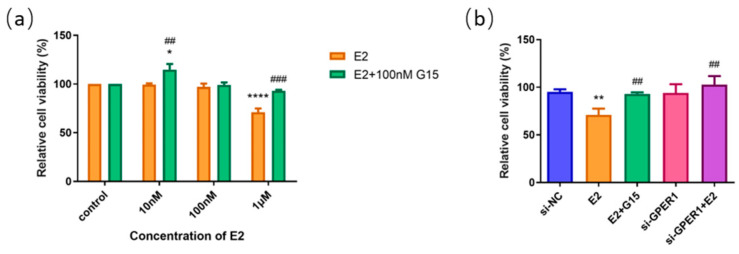
The effect of E2 on the cell proliferation of EC109 cells detected by MTT assay. (**a**) The relative cell proliferation after EC109 cells were treated with different concentrations of E2 and E2 + G15; (**b**) the relative cell proliferation after EC109 cells were treated with si-NC, si-GPER1, and si-GPER1 siRNA + 1 μM G1 group. The results are shown as the mean ± SEM. * *p* < 0.05, ** *p* < 0.01, **** *p* < 0.0001 vs. control/si-NC group; ^##^ *p* < 0.01, ^###^ *p* < 0.001 vs. E2 group.

To identify the functional receptors of E2 on esophageal cell lines, we administered 10 nM, 100 nM, 1 μM E2, and E2 of this concentration gradient with 100 nM G15 to EC109 cells. The results of the MTT assay showed that the proliferation of the EC109 cell line was significantly inhibited with 1 μM E2 treatment alone, which was blocked by G15. However, after using 100 nM G15, the proliferation of the EC109 cell line was promoted with the E2 at low concentration (10 nM) (Figure 7a). To further explore whether E2 affects the proliferation of EC109 cells through activation of GPER1, we knocked down the GPER1 with siRNA and then treated the cells with 1 μM E2. A significant difference was observed between the E2 groups and si-GPER1 + E2 groups in EC109 cells (Figure 7b). These results suggested that E2 suppressed the proliferation of EC109 cells through activation of GPER1.

## 3. Discussion

Malignant tumor esophageal carcinoma exhibits sex bias in terms of incidence, and the sex hormone estrogen and its signaling pathways are considered to play a vital role in the pathogenesis of cancer. The main form of estrogen, E2, could regulate cell activity through binding with ERα and ERβ, and GPER1 in multiple normal and malignant tissues [9,10,29,30]. In the present study, we first explored the transcriptional levels of estrogen receptors (ESR1 and ESR2) and GPER1 RNA in esophageal carcinoma tissues by analyzing the data from GEPIA, which is a web-based tool to deliver fast and customizable functionalities based on The Cancer Genome Atlas (TCGA) and Genotype Tissue Expression project (GTEx) data [31]. The results showed that the RNA levels of ESR1 and ESR2 were not significantly different between esophageal cancer and normal esophagus, and were not correlated with the total survival time of the tumor patients. However, the expression levels of GPER1 RNA were reduced in the overall esophageal cancer tissues compared with the normal esophageal tissues. In addition, low expression levels of GPER1 RNA correlated with reduced survival. The RNA-level analysis of various ERs from the database indicates that estrogen might play a role in the incidence bias of esophageal cancer mainly through GPER1.

Considering that the various subtypes of esophageal cancer have quite different features and epidemiology, we explored the expression levels of GPER1 RNA in different pathologic alterations of the esophagus. There was no subtype division of the esophageal cancer samples in the GEPIA database, while the Oncomine database provides information about different pathological classifications of the diseases [32]. After setting the species, tissues, clinical treatment conditions, and least case number, nine sets of esophageal lesions were selected for analysis. The results showed that three samples of Barrett’s esophagus, one of EAC, and one of ESCC showed overexpression of GPER1 RNA. Barrett’s esophagus is one of the key risk factors and precancerous lesions of EAC [33,34], which prompted us to suspect the presence of various expression characteristics of GPER1 in different histological types of esophageal cancer.

Proteins are functional molecules of the *GPER1* gene. To explore the expression levels of GPER1 in different histological types of esophageal cancer, GPER1 protein were detected in the tissue microarray of EAC, ESCC, and normal controls. Esophageal cancer tissue microarray end products were adopted for uniform staining, which also provided the basic staining pattern of normal tissue to facilitate the standardization of immunohistochemistry results for this study. The results visualized by diaminobenzidine (DAB) showed that the normal stratified squamous epithelia and glandular epithelia in most normal esophageal tissues had a negative or weak expression of GPER1 with light yellow staining. A small partial positive expression of GPER1 was found in some serous cells and gland duct cells, and obvious positive staining was mainly located on the cell membrane of the tumor tissues. Although the cell membrane localization was consistent with the results of a previous study [17], it was also accepted that GPER1 proteins were internalized into the cytoplasm from the cell surface upon ligand stimulation to stimulate the increase in intracellular cyclic adenosine monophosphate or Ca^2+^ concentrations and play functions in intracellular signal transduction [29,35].

Through simultaneous evaluation with the Vectra Multispectral Imaging System, the staining scores for GPER1 demonstrated that there were no obvious differences between the overall esophageal malignant tissues and the normal controls. To further understand the relationship between the expression of this protein and clinicopathological characteristics, we analyzed the GPER1 staining scores of EAC and ESCC, respectively. The results showed that EAC expressed higher levels of GPER1, while ESCC showed relatively lower levels of GPER1 compared with normal esophageal samples, which indicated that the GPER1 protein exhibited a different expression pattern in the subtype of esophageal cancer. This result also helps to explain the existence of the high expression GPER1 RNA in EAC and its low expression in the overall esophageal cancer in the GEPIA database, since ESCC generally accounts for the main incidence of esophageal carcinoma.

According to the positive setting of H-scores, there were no obvious differences between the ESCC and normal control groups for the GPER1 protein. EAC showed an increased ratio of positive staining for GPER1, which was similar to the result directly obtained by the analysis of H-scores. For EAC samples, high expression of GPER1 was associated with poor tumor differentiation. Moreover, positive expression of GPER1 was observed more frequently in the initial stage. Combining the information on GPER1 RNA expression from the Oncomine database in Barrett’s esophagus, we proposed that GPER1 might be correlated with the pathogenic initiation of EAC. Barrett’s esophagus is a precancerous lesion of EAC and is commonly observed in men [36,37,38]. Whether the high expression of GPER1 in Barrett’s esophagus contributes to the transfer of precancerous lesions to malignancy is not currently well known.

Based on the confirmed expression of GPER1 in EC109, we applied this cell line to detect the function of GPER1 in vitro. G1 is the well-known GPER1 selective agonist, and G15 has a similar structure to G1 and is effective in inhibiting all G1-mediated effects [39,40,41]. In the present study, EC109 cells were first treated with G1. We found that G1 suppressed the proliferation and promoted apoptosis through the activation of GPER1 in the EC109 cell line. Nevertheless, the blocking of G15 and silencing of GPER1 with siRNA could effectively reverse and restrain the above effects. Our results are consistent with previous studies showing that activation of GPER1 by G1 exerts an inhibitory effect on various cancer cells [42,43,44]. Previous studies also found that G1 could suppress the proliferation of endothelial cells and breast cancer cells by disrupting microtubule organization during cell mitosis, and the function is independent of GPERs [45,46]. These results indicate that G1 might have various pathways to interfere the cell proliferation in different situations.

E2 was a common ligand for the estrogen receptors ERα, ERβ, and GPER1. Zhang et al. previously found that E2 can inhibit the proliferation of ESCC cells [47]. To investigate whether GPER1 is involved in the E2-induced regulation of cell proliferation, we treated the EC109 cell line with E2 and confirmed that E2 inhibited cell proliferation of esophageal cells. In addition, the inhibitory effect of E2 in the EC109 cell line was reversed and restrained after G15 treatment and GPER1 silencing. These results indicated that the inhibitory effect of E2 on esophageal carcinoma cells might be mediated through the activation of GPER1. Estrogen signals are complex in the human body and vary depending on cell types, subcellular localization, and bound partners of the receptors. Previous studies identified that the ERα could bind to GPER1 and play a regulatory function in inflammation and cell growth [48,49]. Recently, the putative GPER-binding site on the domains of ERα66 and ERα36 was found, which was also considered associated with the clinical therapy effects of tamoxifen for breast cancer [50,51]. The interaction among the GPER1, ER, and ER variants in esophageal cancer requires further study to explore.

## 4. Materials and Methods

### 4.1. Extraction of Data from Databases

The mRNA levels of estrogen receptors, including ERα, ERβ, and GPER1, in esophageal tumor tissues and the clinical information of the patients were extracted from the databases of Gene Expression Profiling Interactive Analysis (GEPIA) http://gepia.cancer-pku.cn/ (accessed on 25 December 2022) and Oncomine http://www.oncomine.org (accessed on 25 December 2022), with GPER1 as the keyword, and the cancer type of esophageal carcinoma was selected.

### 4.2. Immunohistochemistry and Staining Measurement

Esophageal carcinoma tissue microarrays were purchased from Bioaitech Co., Ltd. (Xi’an, China; ethical license: 2005DKA21300), which contained a total of 148 specimens consisting of EAC (n = 50), ESCC (n = 78), and normal esophagus (NE) tissues (n = 20). The streptavidin–peroxidase method was used for immunohistochemistry analysis of the tissue microarrays. The slides were stained with the GPER1 antibody (PA5-28647; Invitrogen, Carlsbad, CA, USA) [12,52,53] and visualized with DAB after incubation with the secondary antibody.

The staining results were analyzed using a Vectra Multispectral Imaging System (Perkin Elmer, Waltham, MA, USA). Vectra 2.0.8 (Perkin Elmer) was used for scanning the slides. After importing the high-magnification images and spectral information in inForm 1.2 (Perkin Elmer), positive cells were identified in the target area and the positive rate was determined by setting the threshold intensity. The expression of GPER1 in the tissue microarray is shown with an automatically calculated H-score (=Σpi*i, where *i* means staining intensity, *i* = 0,1,2,3; *pi* is the percentage of the number of cells with corresponding staining intensity in the overall cells). Positive staining was defined as an H-score ≥ 100 (maximum = 300) in this study.

### 4.3. Cell Culture and Chemicals

The human EAC cell line OE33 and ESCC cell line EC109 were maintained in RPMI1640 (RNBG9150; Sigma-Aldrich, St. Louis, MO, USA) with 10% fetal bovine serum (FBS) (Z7185FBS-500; ZETA^TM^ LIFE, San Francisco, CA, USA) at 37 °C with 5% CO_2_. The telomerase-immortalized human esophageal epithelial cell line NE2-hTERT (NE2) was kindly provided by Professor Shihua Cao (University of Hong Kong) and cultured with a 1:1 mixture of defined keratinocyte serum-free medium (10744019; Gibco, Carlsbad, CA, USA) and EpiLife medium (MEPI500CA; Gibco, Carlsbad, CA, USA) at 37 °C with 5% CO_2_. All the culture media contain phenol red.

GPER1 agonist G1 (10008933; Cayman, Ann Arbor, MI, USA), GPER1 antagonist G15 (14673; Cayman, Ann Arbor, MI, USA), and estrogen E2 (E-2758; Sigma-Aldrich, St. Louis, MO, USA) were dissolved with DMSO and stored at −20 °C according to the company protocol for the treatment of cultured cells.

### 4.4. siRNA Transfection

GPER1 siRNA (si-GPER1, sense GGUGGCCGACUCCCUCAUUTT, and antisense AAUGAGGGAGUCGGCCACCTT) and negative control siRNA (si-NC, sense UUCUCCGAACGUGUCACGUTT, and antisense ACGUGACACGUUCGGAGAATT) were designed and synthesized by GenePharma Co., Ltd., Shanghai, China. The esophageal cells cultured in 6-well plates were transiently transfected with siRNAs using the Lipo8000™ Transfection Reagent (C0533; Beyotime, Shanghai, China) according to the manufacturer’s instructions. The level of GPER1 protein in transfected cells was analyzed by Western blotting to verify the effect of transfection after 48 h.

### 4.5. Western Blot

Western blot analysis was performed with the cell lysates. The cultured cells were harvested and lysed using RIPA buffer (P0013B; Beyotime, Shanghai, China), and then the protein concentration of the supernatant was determined using a BCA Protein Assay Kit (P0012; Beyotime, Shanghai, China). Proteins were separated by electrophoresis in 10% SDS-PAGE gels and transferred to PVDF membranes. The membranes were incubated overnight at 4 °C with a 1:2000 dilution of GPER1 antibody (PA5-28647; Invitrogen, Waltham, MA, USA) or 1:1000 dilution of GAPDH antibody (AF0006; Beyotime, Shanghai, China). After the membranes were washed three times and incubated for 2 h with a 1:1000 dilution of the corresponding HRP-labeled secondary antibody, blotting was detected and visualized with a West Pico PLUS Chemiluminescent Substrate (34580; Thermo Scientific, Waltham, MA, USA).

### 4.6. MTT Assay

Cell viability was assessed using an MTT assay. Cells in 96-well plates were treated with a concentration gradient of G1 or E2 for 48 h in the incubator, followed by the addition of 10 µL of 5 mg/mL MTT to each well for 4 h at 37 °C. After the culture medium with MTT was removed, 150 µL DMSO/well was added, and then the plate was mixed thoroughly on the shaker for 10 min at room temperature. The optical density (OD) was read at 570 nm using the Tecan Infinite M200 Pro Microplate Reader.

### 4.7. Flow Cytometry

Flow cytometry was applied to detect the cell apoptosis. Cells with different concentrations in 12-well plates were collected and suspended in PBS and stained with an Annexin V-FITC/PI Apoptosis Detection Kit (KGA107; Keygen Biotech, Nanjing, China) according to the manufacturer’s instructions. The rate of cell apoptosis was analyzed with Accuri™ C6 Flow Cytometry.

### 4.8. Statistical Analysis

The data from tissue microarray staining were analyzed using GraphPad Prism 6.02 and SPSS 23.0. Differences in the expression levels of GPER1 were assessed using the unpaired *t*-test and Mann–Whitney test. The Pearsonχ² test and Fisher probability test were used to determine the relationship between the positive expression of GPER1 and clinicopathological characteristics. All the cell experiments were repeated three times, and the data were analyzed by the unpaired independent samples *t*-test. The differences were considered statistically significant at *p* < 0.05.

## 5. Conclusions

This study demonstrated that different histological subtypes of esophageal cancer had different GPER1 expression patterns. GPER1 was over-expressed in EAC, while relatively low in ESCC. For ESCC, GPER1 might function as a tumor suppressor by inhibiting proliferation and promoting apoptosis of the tumor cells. For EAC, the role and working mechanism of GPER1 in the disease remain unclear and need to be investigated further in future studies.

## Figures and Tables

**Table 1:** GPER1 expression and clinicopathological data of esophageal adenocarcinoma.

Characteristics		GPER1		*p* Value
		+	−	
		N (%)	N (%)	
Age (years)	≤60	7 (50)	7 (50)	0.429
	>60	23 (63.9)	13 (36.1)	
Sex	Men	28 (59.6)	19 (40.4)	0.651
	Women	2 (66.7)	1 (33.3)	
Differentiation	G1	4 (40)	6 (60)	0.043 *
	G2	14 (58.3)	10 (41.7)	
	G3	12 (75)	3 (25)	
Stage	Ⅰ	5 (100)	0 (0)	0.037 *
	Ⅱ	8 (57.1)	6 (42.9)	
	Ⅲ	10 (43.5)	13 (56.5)	
	Ⅳ	7 (87.5)	1 (12.5)	
Tumor depth	T1	1 (100)	0 (0)	0.175
	T2	10 (83.3)	2 (16.7)	
	T3	12 (48)	13 (52)	
	T4	7 (58.3)	5 (41.7)	
Lymph node	N0	13 (65)	7 (35)	0.783
	N1	9 (52.9)	8 (47.1)	
	N2	3 (50)	3 (50)	
	N3	5 (71.4)	2 (28.6)	

+: H-scores ≥ 100; −: H-scores < 100; *: *p* < 0.05.

## Data Availability

The data supporting the findings of this study are available from the corresponding author upon reasonable request.
